# Progress of Platelet Derivatives for Cartilage Tissue Engineering

**DOI:** 10.3389/fbioe.2022.907356

**Published:** 2022-06-16

**Authors:** Siyu Wu, Wenlai Guo, Rui Li, Xi Zhang, Wenrui Qu

**Affiliations:** ^1^ Department of Hand Surgery, The Second Hospital of Jilin University, Changchun, China; ^2^ Department of Burn Surgery, The First Hospital of Jilin University, Changchun, China

**Keywords:** platelet-rich plasma, platelet-derived growth factor, cartilage tissue engineering, articular cartilage, scaffolds

## Abstract

Articular cartilage has limited self-regeneration ability for lacking of blood vessels, nerves, and lymph that makes it a great challenge to repair defects of the tissue and restore motor functions of the injured or aging population. Platelet derivatives, such as platelet-rich plasma, have been proved effective, safe, and economical in musculoskeletal diseases for their autologous origin and rich in growth factors. The combination of platelet derivatives with biomaterials provides both mechanical support and localized sustained release of bioactive molecules in cartilage tissue engineering and low-cost efficient approaches of potential treatment. In this review, we first provide an overview of platelet derivatives and their application in clinical and experimental therapies, and then we further discuss the techniques of the addition of platelet derivatives and their influences on scaffold properties. Advances in cartilage tissue engineering with platelet derivatives as signal factors and structural components are also introduced before prospects and concerns in this research field. In short, platelet derivatives have broad application prospects as an economical and effective enhancement for tissue engineering–based articular cartilage repair.

## 1 Introduction

Articular cartilage is a highly specialized connective tissue whose function is to provide a smooth, lubricated surface for articulation. As simple tissue without blood vessels, nerves, or lymphatics, it is nourished with oxygen and nutrients diffused from the synovium and the subchondral bone. The total volume of cells in human articular cartilage is only 1.65% on average (Hunziker et al., 2002; Tang et al., 2018), and the rest is made up of the extracellular matrix (ECM), a highly hydrated network of collagens (mostly type Ⅱ collagen) and proteoglycans (Sophia Fox et al., 2009).

Injury to articular cartilage occurs *via* trauma, abnormal wear of an instable joint, excessive load such as sports injuries or obesity, or simply during the aging process. The aforementioned are the main causes of osteoarthritis (OA) and the rapid increase in the incidence of OA, primarily driven by the aging global population, leads to an annually increasing burden on health services and societies worldwide (Quicke et al., 2022).

Due to the less vascularity and restricted number of chondrocytes with weakened proliferative ability and migration, cartilage has a limited capacity for self-repair, especially in large defects (Pearle et al., 2005). Therefore, surgical treatment is necessary, either to accelerate the regeneration or directly fill the defect. Various techniques, including microfracture, autologous chondrocytes implantation (ACI), osteochondral autograft transplantation (OAT), and osteochondral allograft transplantation, have been applied for hyaline cartilage repair. However, the long-term clinical results are not promising. In addition to a higher incidence of complications at the donor site, poor integration between neo-tissue or graft with the native leads to cartilage degeneration and formation of fibrous cartilage that compromises the therapeutic efficacy (Lane et al., 2004; Marcacci et al., 2007; Qi et al., 2009). Cartilage hypertrophy and osteochondrosis have been common adverse reactions of ACI (Niemeyer et al., 2020).

With the growing interest in non-operative treatments and biological adjuncts to surgical treatments to promote cartilage healing and curb degeneration, platelet-rich plasma (PRP), an autologous blood product containing growth factors (GFs), and cytokines have gained significant interest owing to the safety of its autologous origin and relative ease of production. Numerous studies support that PRP has beneficial effects on cartilage pathology and alleviation of pain (Lopez-Vidriero et al., 2010; Fice et al., 2019). It is also been verified to improve the integration of autologous osteochondral grafts at the cartilage interface and decrease graft degeneration, with a raised histological score and few adverse events observed (Smyth et al., 2013).

To overcome the limitations of surgical treatments, such as injury or insufficient supply of the donor site and the potential risk of viral transmission in allograft transplantation, cartilage tissue engineering (CTE) has gained a lot of attention and achievements over years (Zhang B. et al., 2020). Tissue engineering typically involves three main elements: scaffolds, cells, and signaling factors (Langer and Vacanti, 1993). To some extent, the import of biomaterials is a great upgrade of ACI that improves the environment of cell adhesion and anabolism or osteochondral transplantation with less donor site damage. Constructs based on various kinds of biomaterial scaffolds seeded with or without cells have been investigated to improve cartilage repair, for not only maintenance and improvement of the afflicted region but most importantly full regeneration of function (Anderson et al., 2017). Scaffolds are supposed to provide a three-dimensional (3D) normal tissue–mimicking microenvironment for proliferation and differentiation of seeded cells. Ideally, the scaffold used for CTE should be fabricated with biocompatible and biodegradable materials, provide enough mechanical stability during the regeneration process, and support articulate contact. Polymers and decellularized ECM are the most popular materials for CTE scaffolds. Hydrogels composed of highly hydrated polymeric networks possess properties to mimic natural cartilage ECM, such as high water content, biocompatibility, and controllable mechanical strength, and have attracted extra attention for applications in CTE (Yang et al., 2017). Similar to bone tissue engineering, which aims at the diaphysis of long-bones, inorganic materials are also used in the construction of the subchondral bone part of a biphasic or multiphasic scaffold (Bal et al., 2010; Jang et al., 2014; Kosik-Koziol et al., 2020). Another application is to process inorganic materials to nanoscale and incorporate with polymers to create nanocomposite hydrogels for improved bioactivity and mechanical strength (Gaharwar et al., 2014; Qi et al., 2014).

Suitable cells for hyaline cartilage construction include autologous chondrocytes and mesenchymal stem cells (MSCs) obtained from different tissues such as bone marrow, adipose tissue, umbilical cord, synovial membrane or synovial fluid, and chondrogenic progenitor cells (CPCs) (Lee et al., 2013; Wang et al., 2019; Zhang et al., 2020b; Huynh et al., 2021). Although the cellular composition of articular cartilage is rather simple, intercellular communication is still a modulator of cell behavior. A mixture of populations of cells could exceed a single component; as evidence showed, cocultured MSCs and differentiated chondrocytes interact with each other that enhance the functional properties of CTE components and reduce MSC hypertrophy (Bian et al., 2011).

**SCHEME 1:**
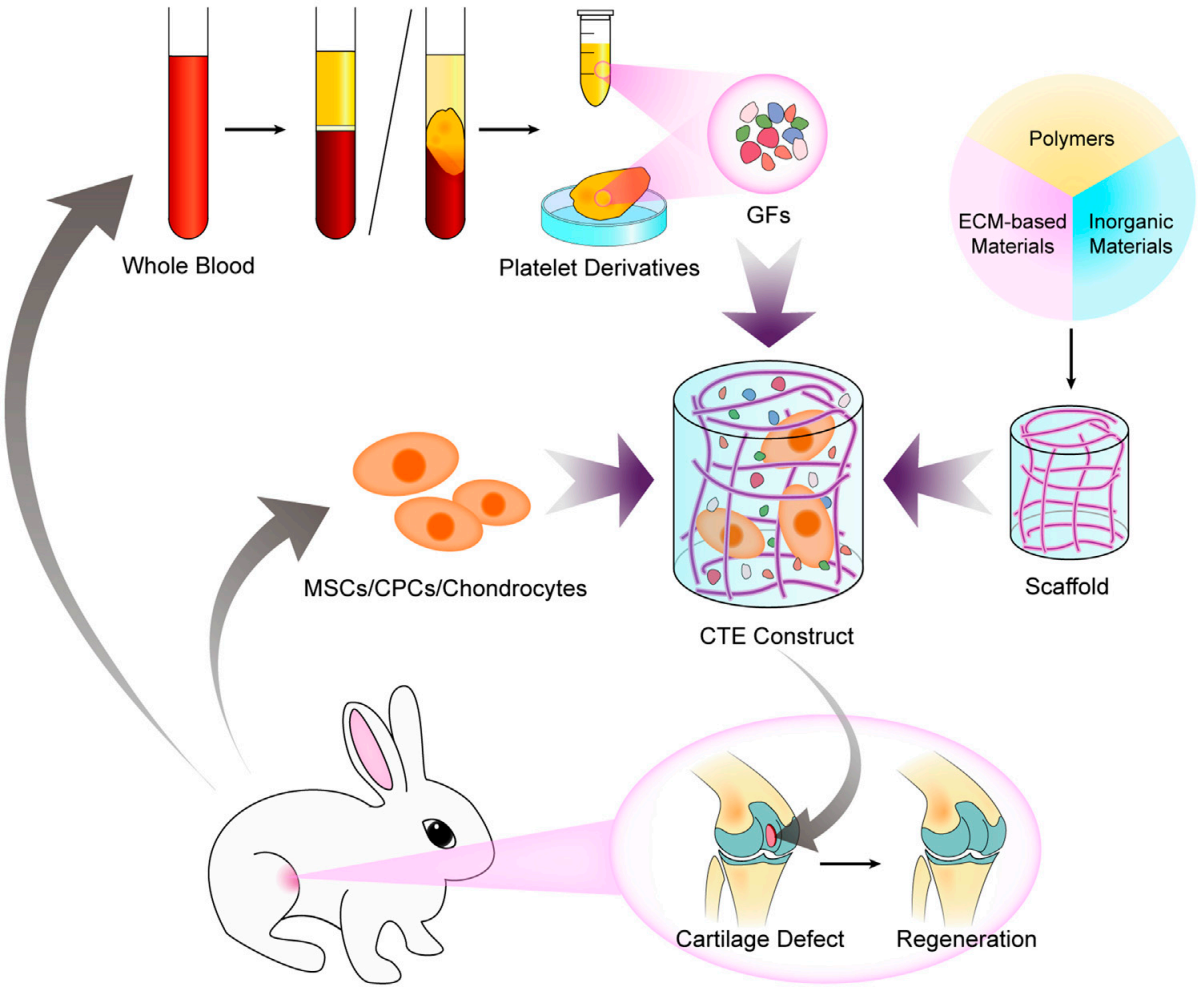
Fabrication of a CTE construct with autologous platelet derivatives, seed cells, and a scaffold for improved regeneration of articular cartilage defects.

The signal factors used in CTE are supposed to mediate growth, proliferation, and differentiation of seeded cells by specific pathway activation and stimulate synthesis and secretion of relevant proteins and glycoproteins for ECM generation and maintenance. Transforming growth factor-β (TGF-β), bone morphogenetic proteins (BMPs), insulin GFs, and fibroblast GFs are among the most popular GFs in cartilage formation (Wei and Dai, 2021). However, recombinant GFs are too expensive for expanded use in clinics, and animal-derived GFs may result in disease transmission or inflammation. Autologous platelet derivatives can be used as a potent and reliable resource of GF cocktail for tissue engineering, as shown in Scheme 1, with low immunogenicity, easy accessibility, and low-cost.

In this review, the characteristics of platelet derivatives and biomaterials, their interaction, and the technology of their combination are discussed. Challenges associated with the use of platelet derivatives in cartilage defects and recommendations for future directions are considered in the final section.

## 2 Platelet Derivatives in Cartilage Repair

Platelets are cytoplasmic fragments of multinucleated megakaryocytes with a life span of 7–10 days, and they play an important role in hemostasis and other aspects. Secretory granules, including ɑ-granules, dense granules, and lysosomes, are critical for normal platelet functions (Frojmovic and Milton, 1982). Among those, α-granules are the most abundant and contain bioactive molecules, including not only GFs and chemokines but also adhesive proteins such as fibrinogen, factors, and enzymes that regulate coagulation and anticoagulation and other basic proteins (Blair and Flaumenhaft, 2009; Foster et al., 2009). GFs, including platelet-derived GF-AB and BB, TGF-β1, vascular endothelial growth factor (VEGF), basic fibroblast GF, epidermal GF, and hepatocyte GF, are stored in α-granules and released after activation (Boswell et al., 2012). The delayed upregulation of insulin GF-1 after PRP use indicates that it regulates local production of these factors (Lyras et al., 2011). PRP and platelet-rich fibrin (PRF) are the most widely used platelet derivatives in clinics, and there are other experimental forms.

### 2.1 Forms of Platelet Derivatives

#### 2.1.1 Platelet-Rich Plasma

PRP refers to a sample of plasma with platelet concentrations above baseline values. One million/ml of platelet count is required based on the working definition, which is approximately five times that of whole blood, based on studies of cell proliferation and tissue healing (Marx, 2001; Dhurat and Sukesh, 2014). It is of a liquid form that is available for injection and coagulation and induced at the same time to prolong the retention.

**SCHEME 2:**
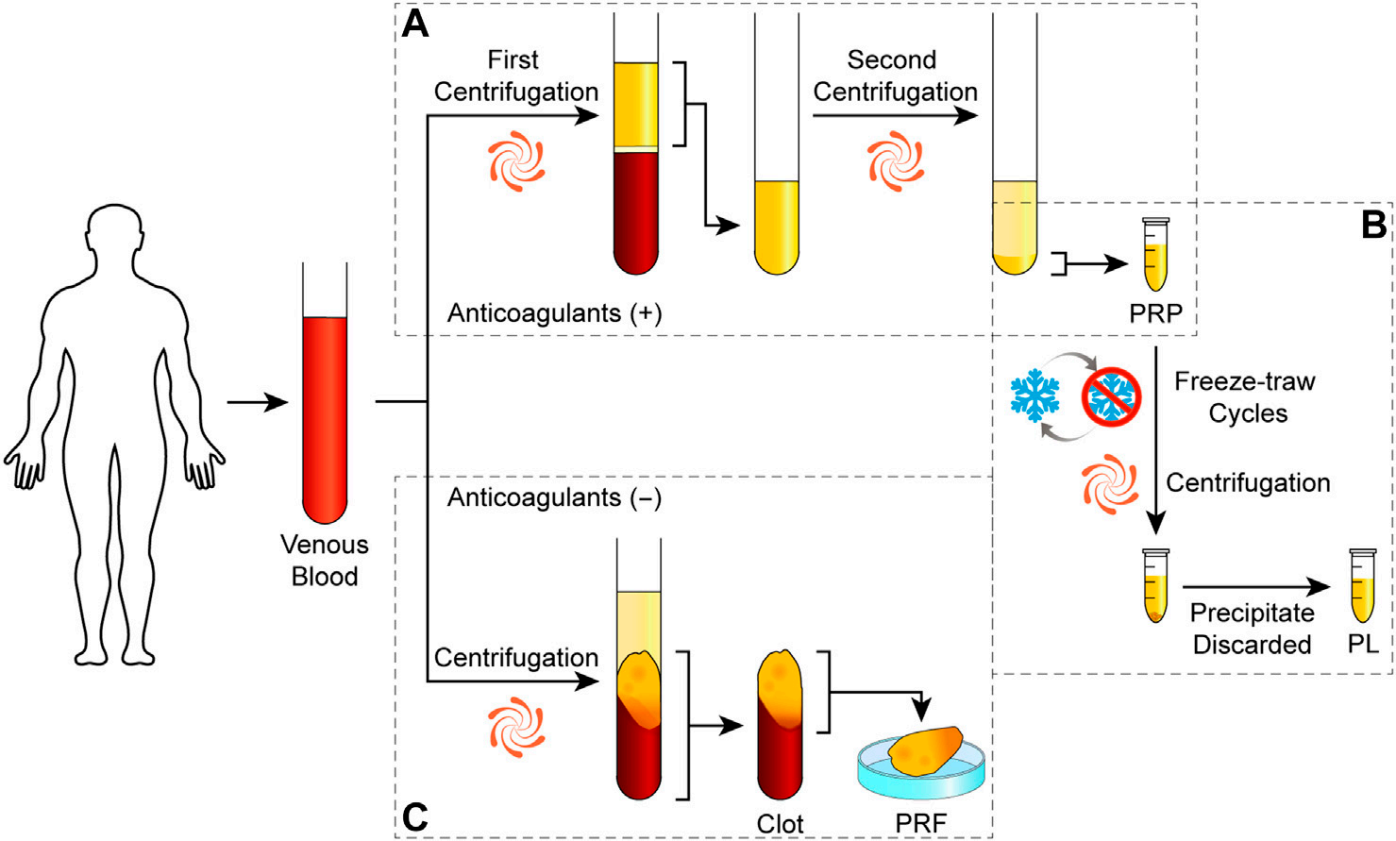
Preparation protocols of platelet derivatives. Schematic drawing of the classical preparation protocols of PRP **(A)**, PL **(B)**, and PRF **(C)**.

As illustrated in Scheme 2A, it can be produced with a double-spin procedure that first reduces the number of erythrocytes and second concentrates platelets, or a single-spin procedure for both purposes. Whole venous blood with anticoagulant undergoes the separating centrifugation. After the first spin, three layers of components are separated: erythrocytes at the bottom, leukocyte-containing buffy coat in the middle, and platelet-containing plasma on the top. Erythrocytes are discarded, while whether to retain the buffy coat leads to different concentration of leukocytes and influences the bio-function of PRP. *In vitro* studies suggest that leukocyte-rich PRP had higher levels of GFs and interleukin-1 receptor antagonist than leukocyte-poor PRP (Cavallo et al., 2014; Ziegler et al., 2019), but their clinical efficacy depends on specific indications (Le et al., 2018; Kim et al., 2021).

#### 2.1.2 Platelet-Rich Fibrin

PRF is the second-generation platelet concentrate derived from PRP. It can be assumed as a piece of hydrogel with a skeleton of fibrin fibers containing platelets and leukocytes. As illustrated in Scheme 2B, the technique requires a blood sample without anticoagulant immediately centrifuged in a glass tube, resulting in a PRF clot in the middle of acellular plasma and a red corpuscle base. It can be transformed into a membrane shape by dry gauze or a compressor for clinical application or cell culture scaffold (Gassling et al., 2010; Kobayashi et al., 2012).

The natural process of production and the absence of anticoagulants or gel agents evaded potential side effects (Dohan et al., 2007). However, the colloidal form could restrict its mixing and combination with materials. Injectable PRF can be prepared with the concept of low-speed centrifugation in a non-coating plastic tube and allows about 80 s for operation before spontaneously clotting (Thanasrisuebwong et al., 2019).

#### 2.1.3 Other Forms of Platelet Derivatives

Bioactive molecules in platelets are also concentrated in other forms for more convenient use. Platelet lysate (PL) is prepared by PRP with two or three freeze–thaw cycles in order to break the platelet membranes and release the bioactive molecules, and the remnants are removed by centrifugation as illustrated in Scheme 2C or by filters (Zaky et al., 2008; Moroz et al., 2013). Platelet-rich concentrate refers to concentrated platelet pellets resuspended in sterile phosphate-buffered saline (Samuel et al., 2019). Plasma with anticoagulant is removed such as washing red cells, which may reduce potential adverse immune reactions when using allogenic resources (Akbarzadeh et al., 2021). Aggregates can be removed by centrifugation after the induced degranulation, which leaves only soluble components for application (Gruber et al., 2002; Verrier et al., 2010). It indicates the possibility to use apheresis platelet concentrates for less erythrocyte waste or blood bank–pooled platelets that might be discarded through blood donation services.

Platelet-derived extracellular vesicles including exosomes and microvesicles are regarded as important effectors of platelet derivatives for encapsulation of bioactive content and enrichment of micro RNAs (Torreggiani et al., 2014; Johnson et al., 2021). Their isolation from activated PRP or PL requires more complicated methods such as differential centrifugation, ultracentrifugation, tangential-flow filtration, and size-exclusion chromatography and also leads to waste of free molecules released from platelets (Aatonen et al., 2014; Torreggiani et al., 2014; Gomes et al., 2022). Despite the need of production optimization, extracellular vesicles showed a potential way to extract or eliminate specific subsets of platelet-derived bioactive molecules through different stimulus or by the characteristic of extracellular vesicles (Aatonen et al., 2014; Ferreira et al., 2020). Therefore, extracellular vesicles, as an important carrier of cellular paracrine, have a phospholipid bilayer membrane structure, contain a large number of bioactive molecules, and participate in the development and repair of important organs; the application of platelet-derived extracellular vesicles in CTE has great potential. For the aforementioned reasons, its therapeutic effect needs to be further verified.

Regarding the acting mechanism of platelet derivatives, the most current researches on their promotion of cartilage regeneration stayed at the level of therapeutic effect, and the mechanisms behind are rarely studied because of their complex composition. We believe that the in-depth mechanism research will effectively promote the progress of platelet derivatives in CTE.

### 2.2 Activation of Platelet Derivatives

Activation of platelet concentrates leads to not only the release or derivation of stored GFs and cytokines but also rapid translation of preexisting mRNA (Senzel et al., 2009). Typically, exogenous thrombin and/or calcium should be added to PRP for platelet activation and coagulation for the initial burst release of GFs, followed by further sustained synthesis and secretion for the remaining several days of their life span (Freymiller, 2004). The contact with a silica surface activates platelets in PRF and the coagulation cascades, while trapping GFs and cytokines in the fibrin network for prolonged life span and slow release (Dohan et al., 2006; Ehrenfest et al., 2012). Bioactive molecules can also be released by simply breaking the membrane of platelets during the production of PL or by mechanical force through a 0.45-μm syringe filter (Huynh et al., 2021).

**FIGURE 1:**
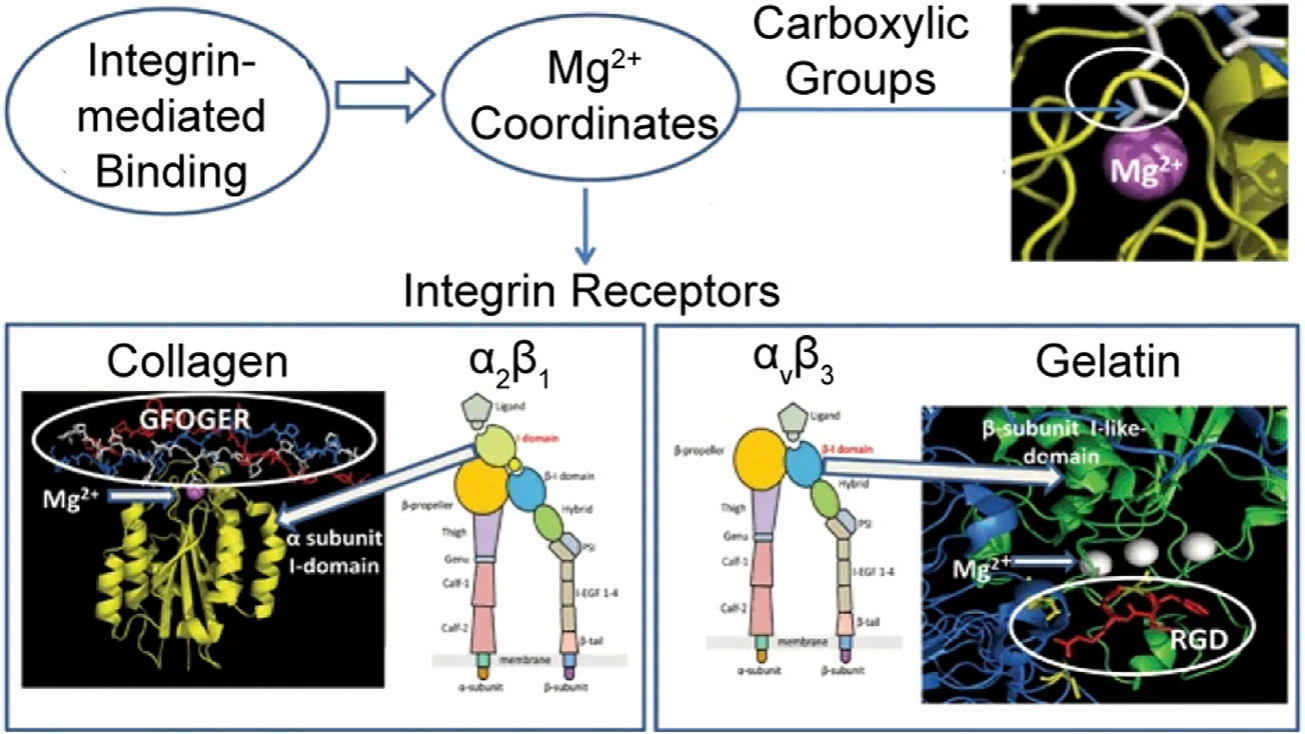
Graphical representation of integrin-mediated adhesion on collagen and gelatin. Reproduced with permission from Davidenko et al. (2016).

Alternatively, local tissue can mediate endogenous release that results in a slower release of GFs and other chemical mediators (Mehta and Watson, 2008). The integrin signaling pathway of platelet activation is firstly recapitulated by Qureshi et al. from a protein–protein interaction network, as shown in Figure 1 (Qureshi et al., 2009). Platelets can be activated *via* connection to integrin receptors on collagen or gelatin that initiate the degranulation and the release of GFs without additions or mechanical forces (Davidenko et al., 2016). Other approaches, such as thrombin receptor agonist peptide, showed less clot retraction and delayed release of GFs (Landesberg et al., 2005). Irmak et al. realized photo-activation of PRP by polychromatic light in the near-infrared region (PAC) and a constant release of GFs for several weeks (Irmak and Gumusderelioglu, 2020).

There are many available commercial platelet-rich concentrations producing systems with various platelet, white blood cell (WBC), and GF content (Castillo et al., 2011; Kushida et al., 2014), and the convenience of production and extensive use in clinics showed mutual promotion to the maturity and spread of the procedure.

### 2.3 Biological Function of Platelet Derivatives

Platelet derivatives have been applied in fields including wound healing, dental and oral surgery, and sports medicine (Crovetti et al., 2004; Lopez-Vidriero et al., 2010; Albanese et al., 2013). There are numerous clinical objectives, including promotion of tissue regeneration, prevention, or treatment of infectious diseases and pain relief (Bibbo and Hatfield, 2010; Drago et al., 2013; Drago et al., 2014; Chen et al., 2018).

PRP has been reported to significantly downgrade the expression of inflammation-induced cytokines and chemokines in chondrocytes, thus preserving proliferation and chondrogenic phenotypes reduced by pro-inflammatory cytokines with or without the enhancement by hyaluronic acid (HA) (Chen et al., 2014). Although a concentration of IL-1β is detected in some platelet derivatives, which is considered a defect of regeneration (Sundman et al., 2011), its rich in chemokine release upregulation of MSCs could reduce OA pathology through attraction and phagocytosis enhance of polymorphonuclear cells (Van Dalen et al., 2019).

**FIGURE 2:**
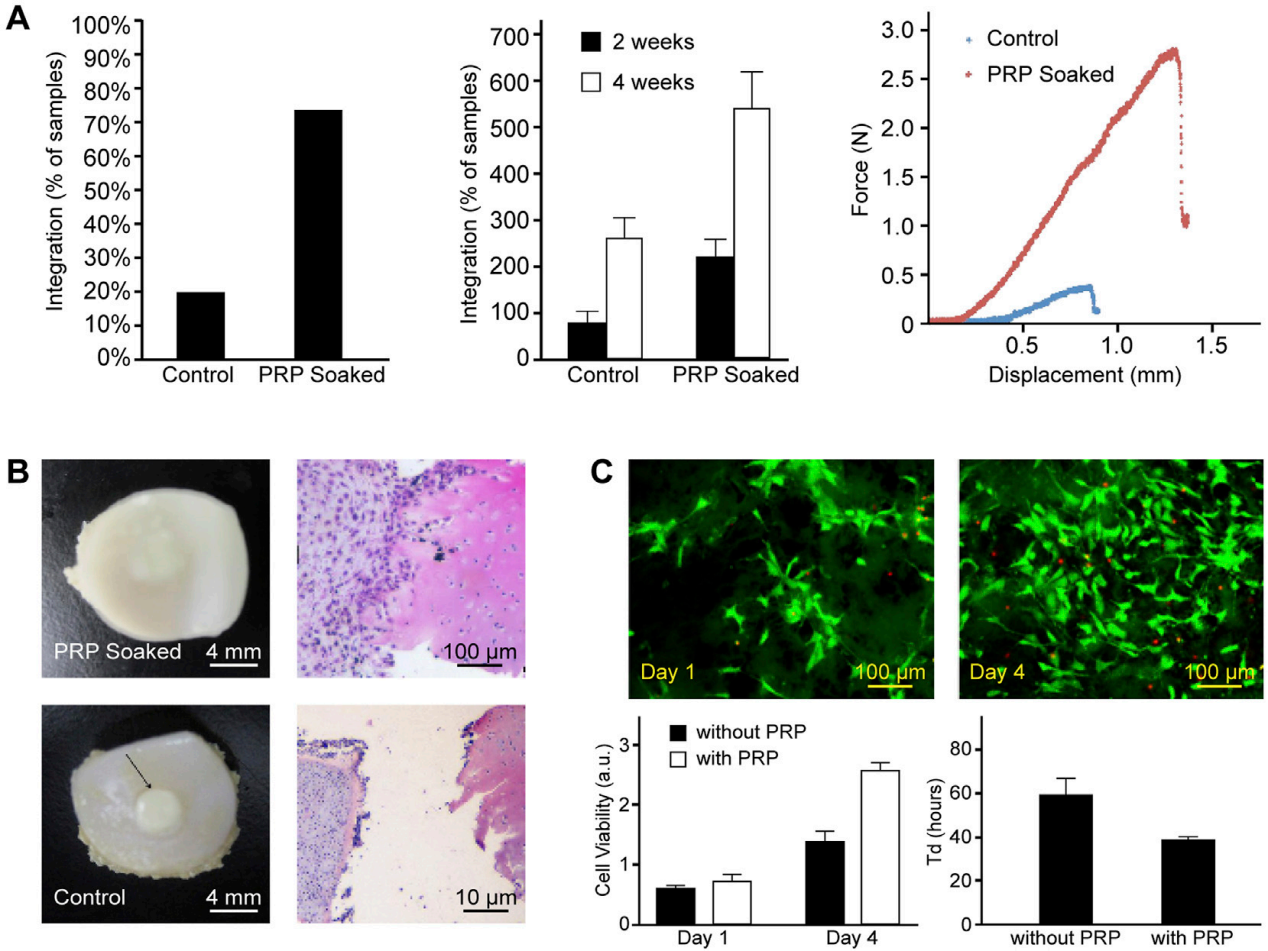
Encapsulation of PRP enhances integration of implants with native tissue and MSC proliferation on scaffolds. **(A)** PRP-soaked bioengineered implants showed a higher percentage and greater strength of integration. **(B)** Gross and histological appearance of PRP-soaked and untreated implant–explant constructs. Reproduced with permission from Sermer et al. (2018). **(C)** Fluorescence images by LIVE/DEAD staining. Statistical results showed enhanced cell viability and a shortened doubling time of MSCs in the parylene-based scaffold when compared to the sample group without PRP. Reproduced with permission from Wu et al. (2020a).

In cartilage repair, platelet derivatives increase chondrocyte viability, proliferation, and migration (Chien et al., 2012; Fice et al., 2019). Chondrocytes cocultured with platelets showed significantly increased production of BMP-7, which upregulates proliferation by autocrine or paracrine and accelerates the appearance of hyaline-like tissue (Hidaka et al., 2003; Zhou et al., 2016). They enhance phenotype maintaining sulfated glycosaminoglycans (GAGs) secretion for ECM accumulation resulting in the presence of better mechanical properties (Akeda et al., 2006; Petrera et al., 2013; De Angelis et al., 2020). As shown in Figures 2A,B, implants soaked in PRP showed better integration to native tissue with both mechanical and histological evidence (Sermer et al., 2018; Wu C.-Y. et al., 2020). Furthermore, PRP induces redifferentiation of dedifferentiated chondrocytes (Jeyakumar et al., 2017).

Platelet derivatives showed similar promotive influences to MSCs too, as shown in Figure 2C (Mishra et al., 2009; Van Pham et al., 2013). Zaky et al. suggest conspecific PL being an effective and more beneficial substitute of fetal bovine serum to support the *in vitro* expansion of human bone marrow mesenchymal stem cells (BMSCs) for tissue-engineering applications based on findings that BMSCs cultured in media with PL and deprived of serum showed a high proliferation potential and formed organized ECM with embedded chondrocytes in lacunae (Zaky et al., 2008). Platelet-derived microparticles, fragments shed from the plasma membrane of activated platelets, were found in contact with synovial membrane–derived MSCs and promote their homing and adhesion to cartilage defects (Liang et al., 2020). In addition, platelet can elicit chemotaxis and chemokinesis of MSCs, thus inducing recruitment of endogenous joint-resident MSCs and serve the regeneration purpose (Holmes et al., 2018; Zhang S. et al., 2020). Therefore, platelet derivative–loaded scaffold without seeded cells can still induce the repair of cartilage defects (Sun et al., 2010; Liu et al., 2015), and in some circumstance, additional MSCs are redundant (Zhang et al., 2015).

Previous prevailing views suggested that GFs are the main mediators of platelet derivatives’ regenerative effects, and they are adopted as important characterization parameters second only to cell count (Fernandez-Medina et al., 2019; Oneto et al., 2020). With further insight into exosomes and their role in intercellular communication, it is also revealed that the packaged non-coding RNAs also contribute to their treatment of OA (Maehara et al., 2021; Puhm et al., 2021).

The mechanisms of GFs and micro RNAs in cartilage regeneration have been studied individually and thoroughly reviewed in the literature (Qian et al., 2017; Barbon et al., 2019; Chen et al., 2020). However, as biologics are complex mixtures, the biological action of platelet derivatives is due to the synergistic action of the components, and the crosstalk, synergism, or mutual antagonism between signaling pathways is unevaluable. Therefore, the underlying mechanism remains partially covered. In addition, their composition and efficacy are influenced by many factors, such as the extraction protocols, storage conditions, physical status, and gender of the donor (Evanson et al., 2014; Do Amaral et al., 2016; Yin et al., 2017; Fernandez-Medina et al., 2019; Kim et al., 2020; Delgado et al., 2021).

Standardization of platelet derivatives is urgently needed, although their biological origin ensures variability to some extent. Additional information is required to maximize reproducibility of results, not only characterizations of the final product, including detailed cell counts and quantities of representative GFs, but also protocols as detailed as a needle size used for drawing blood, temperature, and time from preparation to use (Fadadu et al., 2019). A standardized preparation protocol for robust production and an optimized dosage needs to be determined. However, it should be noted that the detailed characterization should be done in clinic only for research purposes rather than on every patient, as it violates the original intention of reducing the cost, and that may explain the insufficiency of product data for previous retrospective studies (Landi et al., 2011).

## 3 Addition of Platelet Derivatives in CTE

The addition of platelet derivatives mainly serves two purposes. The first and most important is the rich biomolecules, such as GFs, it contains. The second is the fibrinogen and gelling ability after activation as all or part of the scaffold structure.

**FIGURE 3:**
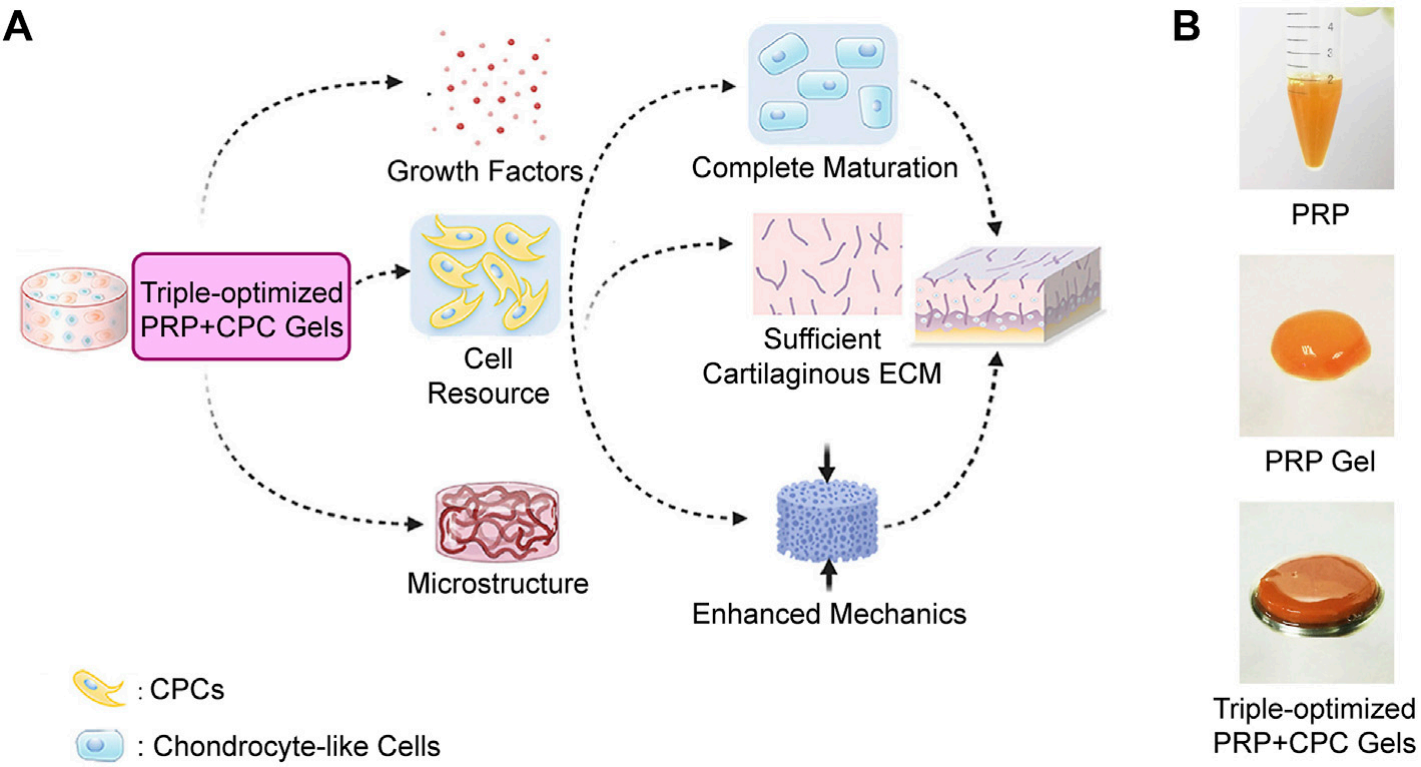
Cellular, biochemical, and biomechanical triple-optimized PRP + CPC constructs enhance cartilage regeneration. **(A)** Schematic of the optimization strategy and potency of cartilage regeneration. **(B)** Gross appearance of PRP, PRP gel and triple-optimized PRP + CPC construct. Reproduced with permission from Wang et al. (2021).

Platelet derivatives act as a bioactive cell scaffold to fill defects and enhance cartilage regeneration. The fibrin matrix not only forms a 3D system for cell culture but also reserves biomolecules with binding domains and releases them gradually during degradation (Xie et al., 2012; Martino et al., 2013). The mechanical stiffness of the fibrin clots depends upon the concentration of agonist (thrombin, calcium, fibrinogen, etc.), fibrin network, and crosslinking densities. For better mechanical properties, 10% CaCl_2_-40 U/ml bovine thrombin in 1:9 v/v was recommended by Wang et al. and used as the scaffold for CPCs culture and cartilage defect repair *in vivo* (Figure 3) (Wang et al., 2021). Though the matrix of fibrin showed limited preservation of GFs, the release was significantly slowed down after 2 days (Liu et al., 2017). In fact, thrombin-activated platelets degranulate and release nearly 100% of the GFs within an hour (Marx, 2001). Moreover, its rapid degradation *in vivo* or with enzyme presents and low mechanical stiffness compared to articular cartilage expand the limitations of the scaffolds made of platelet derivatives alone (Lucarelli et al., 2010; Isobe et al., 2017; Huynh et al., 2021), while the integration with biomaterials will possess the scaffold of more controllable mechanical properties, proper degradation rate, and long-term release of bioactive molecules such as GFs (Sell et al., 2011; Bolandi et al., 2021).

### 3.1 CTE Scaffold With Platelet Derivatives

In this part of the review, the designs of platelet derivative–based CTE constructs are discussed by fabrication and composition. The designs of CTE constructs with addition of platelet derivatives in the scaffolds are summarized in Table 1.

**TABLE 1:** Designs of CTE constructs with platelet derivatives in the scaffolds.

Category	Material	Design	Platelet derivatives	Activation method	Loading method technique	GFs reservation	Cell *in vitro*	Cell *in vivo*	References
ECM-based materials	Decellularized human placental	Solid scaffold	PRP	Integrin pathway	Immersion	Adsorption GF–binding domains	BMSCs	BMSCs	Ozdemir et al. (2020)
	Decellularized porcine cartilage	Microparticle + PRP Gel	PRP	Ca^2+^ and thrombin	Mixing and clotting	Adsorption GF–binding domains	Chondrocytes	Chondrocytes	Chen et al. (2021)
Natural polymers	Collagen	Bilayer solid scaffold	PRP	Integrin pathway	Dripping	Adsorption	N/A	None	Qi et al. (2009)
	Fibrin	Hydrogel	PRF exudates	Coagulation	Mixing	Physical encapsulation GF–binding domains	Chondrocytes	N/A	Chien et al. (2012)
	SF	Solid scaffold	PRP	None	Mixing	Physical encapsulation	Chondrocytes	N/A	Li et al. (2020)
	Agarose	Hydrogel	PRP	None	Mixing	Physical encapsulation	Chondrocytes	N/A	Yin et al. (2014)
	Algae	Injectable hydrogel	PRP	Ca^2+^	Mixing	Adsorption Physical encapsulation	BMSCs	N/A	Gao et al. (2019)
	Gel MA	Triphasic 3D-printed hydrogel	PRP	Polychromatic light	Mixing	Adsorption Physical encapsulation	AdMSCs	N/A	Irmak and Gumusderelioglu (2021)
	Gel MA	3D-printed hydrogel	PRP	Integrin pathway	Mixing	Adsorption Physical encapsulation	BMSCs	None	Jiang et al. (2021)
	Gel MA	3D-printed hydrogel	PRP	Ca^2+^	Coating	Attaching Adsorption	BMSCs	BMSCs	Luo et al. (2020)
	Gelatin–PEG–tyramine	Injectable hydrogel	PRP	Integrin pathway	Mixing	Adsorption Physical encapsulation	Chondrocytes	Chondrocytes	Lee et al. (2012a), Lee et al. (2012b)
	HA–tyramine	Injectable hydrogel	PL	Lysis	Mixing	Adsorption Physical encapsulation GF–binding domains	BMSCs	N/A	Jooybar et al. (2019)
	HA-o-nitrobenzyl alcohol	Injectable hydrogel	PRP	Integrin pathway	Mixing	Adsorption Physical encapsulation GF–binding domains	BMSCs	None	Liu et al. (2017)
	Chitosan/CS	Injectable nanoparticle hydrogel	PL	Lysis	Mixing	Adsorption GF–binding domains	AdMSCs	N/A	Santo et al. (2015)
	HA/CS/chitosan	Hydrogel	PRP	Integrin pathway	Mixing	Adsorption Physical encapsulation	AdMSCs	N/A	Khalilijafarabad et al. (2021)
	Fibrin/heparin/linker peptide	Hydrogel	PRP	Thrombin	Mixing	Physical encapsulation GF–binding domains	BMSCs	N/A	Ahmed et al. (2011)
Synthetic polymers	PLGA	Solid scaffold	PRP	Ca^2+^ and thrombin	Immersion-clotting	Adsorption	BMSCs	BMSCs	Tang et al. (2021b)
	PLGA	Bilayer solid scaffold	PRP	Ca^2+^	Immersion	Adsorption	BMSCs	BMSCs	Zhang et al. (2015)
	PPX	Solid scaffold	PRP	None	Mixing	Physical encapsulation	AdMSCs	N/A	Wu et al. (2020a)
	PCLT–citrate	Solid scaffold	PRP clot exudates	Ca^2+^	Immersion	Adsorption	Chondrocyte	N/A	Rothan et al. (2017)
	Dextran–tyramine	Injectable hydrogel	PL	Lysis	Mixing	Adsorption Physical encapsulation	BMSCs/chondrocytes	N/A	Teixeira et al. (2012b)
	PCL/gelatin	Solid scaffold	PRP	Ca^2+^	Mixing	Adsorption Physical encapsulation	BMSCs	None	Liu et al. (2015)
	Alg sulfate, PVA/Alg	Microparticle + Injectable hydrogel	PRP	Ca^2+^	Mixing	Adsorption Physical encapsulation	AdMSCs	N/A	Bolandi et al. (2021)
	EPL/Heparin, PLEL	Nanoparticle + Injectable hydrogel	PL	Lysis	Mixing	Adsorption Physical encapsulation GF–binding domains	Chondrocytes	None	Tang et al. (2021)
	PCL/chitosan/PEG-biotin, collagen/fibrin	Microparticle + Hydrogel	Platelet-rich concentrate	Ca^2+^ and thrombin	Mixing	Physical encapsulation GF–binding domains	Chondrocytes	None	Filova et al. (2020)
Inorganic materials	Calcium polyphosphate	Solid scaffold	PRP	None	Immersion	Adsorption	Chondrocytes	N/A	Sermer et al. (2018)
	β-TCP/gelatin	Biphasic Solid scaffold	PRP	Integrin pathway	Dripping	Adsorption	BMSCs	BMSCs	Seo et al. (2013); Seo et al. (2015)
	HAP/fibrin	Biphasic Solid scaffold	PRP	Ca^2+^ and thrombin	Immersion-clotting	Adsorption GF–binding domains	BMSCs	BMSCs	Jang et al. (2014)
	Nano-HAP/chitosan/SF	Solid scaffold	PRP	None	Dripping	Adsorption	BMSCs	BMSCs	Ruan et al. (2018)

Abbreviations: N/A, not applicable; PCL. poly(ε-caprolactone); PVA, poly(vinyl alcohol); EPL, ε-poly(L-lysine); PLEL, poly(d,L-lactide)-poly(ethylene glycol)-poly(d,L-lactide); PPX, poly(p-xylylene); PCLT, poly(caprolactone triol).

#### 3.1.1 Design of CTE Constructs From Application Point of View

When compared to the surgical options in clinic, the CTE constructs can be cultivated into artificial hyaline cartilage *in vitro*, trimmed, and transplanted into the defect as an improved OAT with sufficient graft but no donor site damage. With the help of developed bioreactors, automatic nutrition, standardized oxygen concentration, dynamic mechanical stimulation, and other improvements are applied for high-quality artificial cartilage (Guo et al., 2016; Fu et al., 2021). In addition, the condition of neo-cartilage formation is available for histological examination and non-invasive monitoring such as an ultrasonic signal analysis (Melchor et al., 2018). Otherwise, the cell-laden scaffolds can be implanted right after the fabrication as scaffold-assisted ACI with better cell adhesion and anabolism and form neo-cartilage internal. The procedure is simplified and fewer devices are required.

Both open surgery and arthroscopic surgery are available for the implantation of CTE construct. The arthroscopic procedure fits the cosmetical pursuit of the patients. On the contrary, the open procedure provides sufficient exposure and direct access to intra-articular lesions and thus has advantages over arthroscopy when the defect is large or in restricted areas (Kizaki et al., 2021).

From the perspective of form, the CTE construct can be either solid or liquid before implantation, which is described as injectability.

##### 3.1.1.1 Uninjectable Constructs

Strategies for uninjectable CTE constructs include those use solid scaffolds or injectable formula but are cultivated *in vitro* for graft. Various mature and innovative techniques are used for solid scaffold fabrication, such as solvent casting/particulate leaching, melt molding, gas foaming, phase separation, freeze drying, electrospinning, and additive manufacturing (Mabrouk et al., 2020). In addition to the highly control over the scaffold structure and evaluation of neo-tissue, uninjectable strategies easily realize the manufactory of multiphasic scaffolds to satisfy different mechanical and biological requirements in both cartilage and subchordal bone layer for cell specialization (Yousefi et al., 2015). The addition of platelet derivatives will be discussed in a later section of this article.

*In situ* assembly of CTE constructs with several commercialized materials is another option for convenient clinical use. The platelet derivatives are used as both GF-resources and structural components that hold everything together. HA-derivative felt, PRP or PRF, and cartilage fragments are orderly assembled as cell resource in the defect site with the bridge reinforced by additional fibrin glue (Marmotti et al., 2012; Marmotti et al., 2013). In the original hypothesis, cartilage harvesting should be performed right before implantation in the same operation, thus eliminating the effort and cost of cultivation (Marmotti et al., 2012). Though the sufficiency of chondrocyte is suspicious, the result supports the strategy which is closer to the clinic.

##### 3.1.1.2 Injectable Constructs

The advantage of injectable CTE constructs is obvious: In addition to the cooperation with micro-invasive surgery, the initial fluidity allows conformal filling of irregular defects. The constructs are typically processed into small particles or *in situ* crosslinking hydrogels. As cartilage kibbling is a necessary procedure to minimize the time of chemical exposure during decellularization, ECM-based materials are sometimes present as particles available for injection (Sutherland et al., 2015). The addition of platelet derivatives synergizes the particle strategy as the coagulation entraps the particles in fibrin network and fixes them to the defect. The fixation and biophysical modification could also be realized by embedding in hydrogels (Williams et al., 2015; Raj et al., 2020). Chen et al. made a 3D semisolid gel of a mixture of decellularized porcine cartilage, chondrocyte suspension, and freshly activated PRP for *in vitro* and *in vivo* evaluation (Chen et al., 2021). The composite mimics the natural niche for chondrocytes and accelerated the regeneration of full-thickness osteochondral defects in mini-pigs. A similar system without cell transplantation was used through intra-articular administration in a rat OA model and proved efficient in cartilage repair (Wu et al., 2021).

As for injectable hydrogels, both physical and chemical crosslinking reactions are applicable. The former is generally induced in milder conditions with fewer toxicity problems, and the latter provides more stable networks and better mechanical properties (Wu J. et al., 2020). Ion-induced cross-linking is the most compatible physical cross-linking method for platelet derivatives as the most frequently used ionic agent, CaCl_2_, also induces assembly of fibrinogen to form an interpenetrating polymeric network with the material that modulates properties of the hydrogel (Faramarzi et al., 2018). Thermosensitive hydrogels that are present in the sol state at room temperature and gels at body temperature is used for PL-conducting nanoparticles delivery (Tang Q. et al., 2021).

**FIGURE 4:**
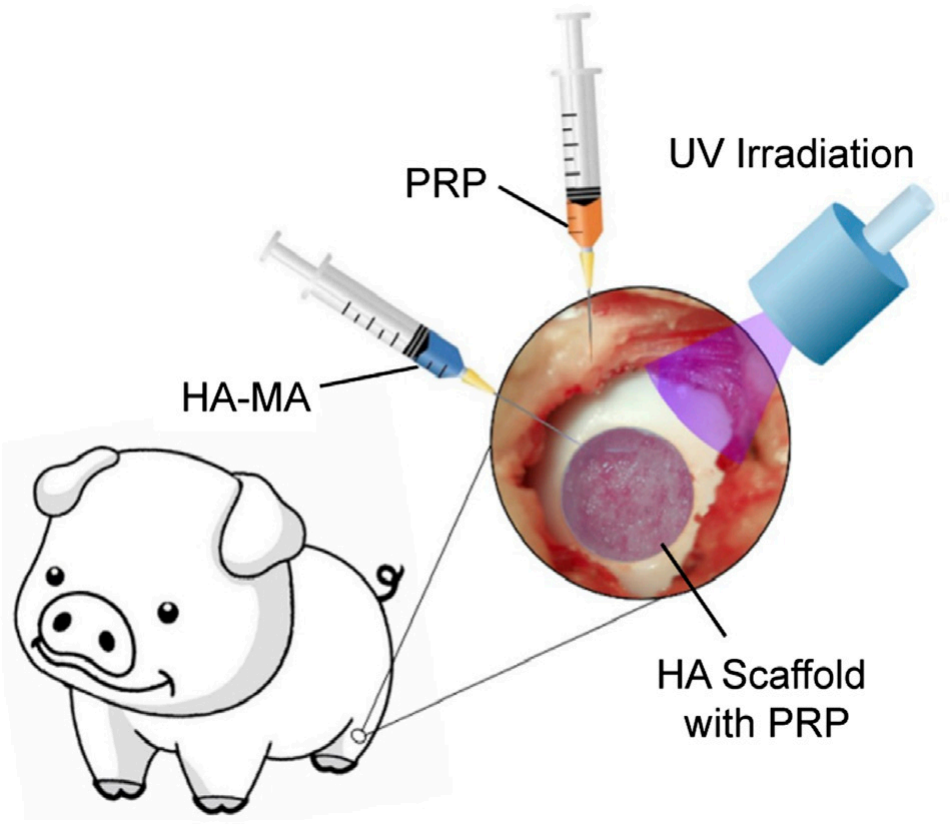
Schematic of the surgical procedure for implantation of photo-cross-linked injectable scaffolds. Reproduced with permission from Yan et al. (2020).

Photo-crosslinking is a widely used chemical crosslinking strategy for *in situ* gelation. As illustrated in Figure 4, the procedure of surgery includes debridement of the lesion, application of liquid materials, and ultraviolet irradiation. Liu et al. developed an *ο*-nitrobenzyl alcohol–modified HA hydrogel system, based on the photoinduced imine crosslinking reaction (Liu et al., 2017). Under light irradiation, aldehyde groups are generated that subsequently react with amino groups distributed on fibrinogen in the components of PRP and tissue surface, which forms hydrogels *in situ* and synchronously realizes covalent hydrogel-tissue adhesiveness and integration. In addition, the GFs were almost linearly released with no trend of decline shown in the release of GFs even after 14 days. Enzymatic crosslinking is another commonly used option for injectable application strategy of functionalized polymers that circumvents ultraviolet-induced free radicals (Klotz et al., 2016). Gelation of polymers with tyramine moieties can be catalyzed within minutes by horseradish peroxidase at very low concentrations of H_2_O_2_, and the injectable hydrogel system is used for local delivery of platelet derivatives and seed cells for chondrogenesis *in vitro* and *in vivo* (Lee et al., 2012a; b; Jooybar et al., 2019). Horseradish peroxidase also enables covalent crosslinking between tyramine groups on polymers and tyrosine-containing cartilaginous ECM proteins. Teixeira et al. fabricated a dextran–tyramine injectable hydrogel with a sustained release of PL. During *in situ* gelation, covalent bonding of the hydroxyphenyl residues in the material to tyrosine residues in cartilage matrix proteins enhances the adhesion and retention of the hydrogel, thus improving the integration of the newly regenerated tissue with native cartilage (Teixeira et al., 2012a; Teixeira et al., 2012b).

#### 3.1.2 Techniques of Addition and the Influences on Scaffold Proprieties

On a micro level of most strategies, GFs are directly attached to biomaterial surface by noncovalent methods, including adsorption, physical encapsulation, or ECM components with GF-binding domains such as heparin, chondroitin sulfate, and hyaluronic acid (Seims et al., 2021). Hydrogen bonding, electrostatic interaction, and pore filling effect hold the bioactive molecules to the surface of material (Wang et al., 2020). Compared to covalent methods, fewer agents or linkers are involved and disruption of GF conformation is minimized. In addition, the molecular complexity of platelet derivatives greatly increases the difficulty of covalent techniques. On the other hand, fibrin/fibrinogen, component of platelet derivatives, shows high affinity for various kinds of GFs, and the promiscuously bound through heparin-binding domain will prolong the retention of GFs (Martino et al., 2013). Thus, compared to single GF-conducting, the combination of platelet derivatives and biomaterial is much simpler as both direct reservation of GFs and the tangle with fibrin can serve the purpose of a sustained release.

On a macro level, the combination of platelet derivatives and biomaterials is much simpler based on their solid or liquid form and technology roadmap. As the porous structure of scaffolds is necessary for cell ingrowth and nutrition transport, plenty of solid scaffolds possess the ability to absorb and contain liquids such as a sponge and hydrogels can take in soluble molecules during swelling (Hollister, 2005). Most of the platelet derivatives are presented in liquid form, thus the easiest way to load them onto molded scaffolds is by soaking (Rothan et al., 2017; Sermer et al., 2018). Additional small pores were discovered on the scaffold surface after PRP treatment and may improve cell adhesion (Gentile et al., 2010; Wei et al., 2019).

**FIGURE 5:**
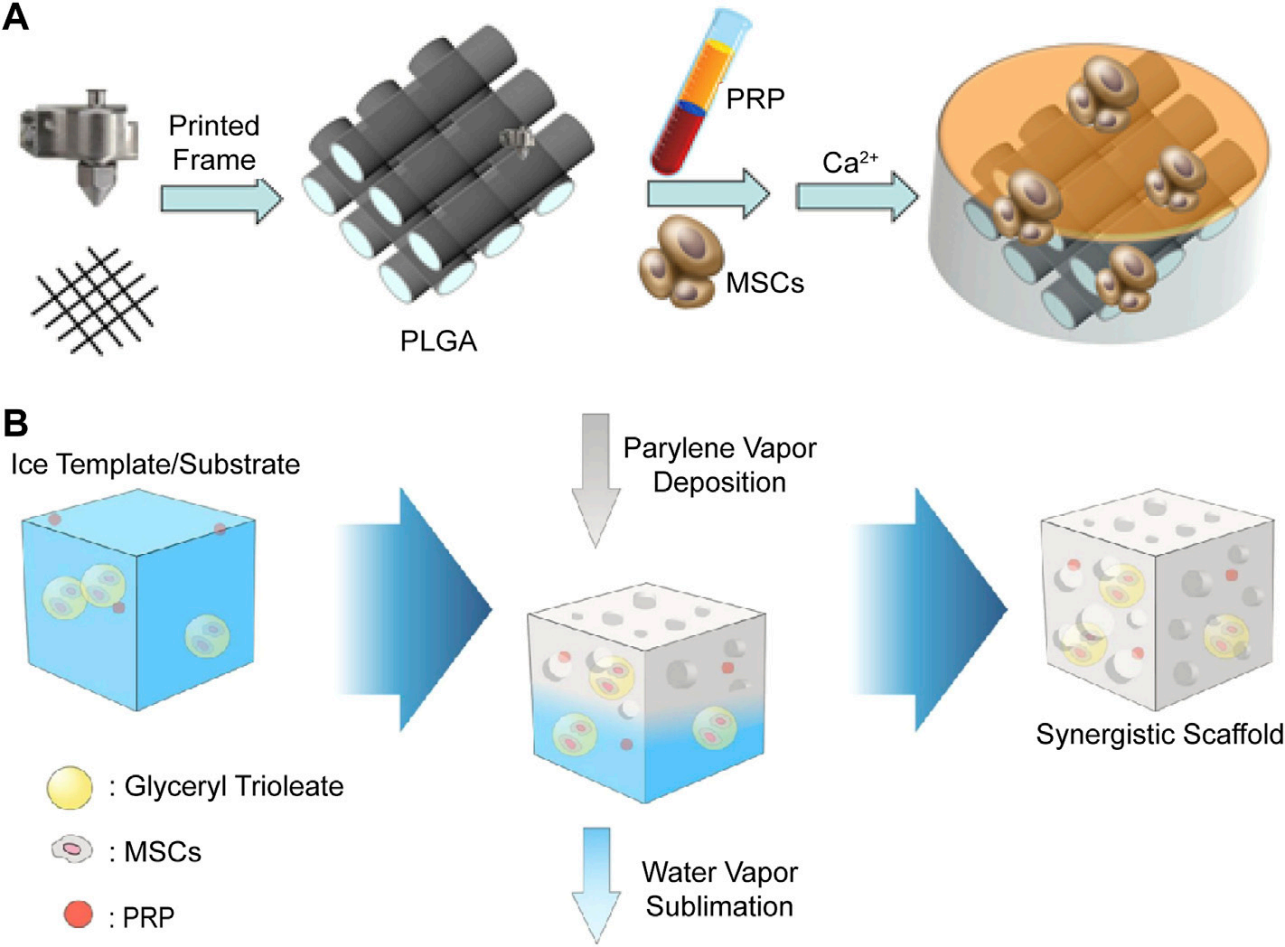
Schematic illustration of two methods to add platelet derivatives into the scaffolds. **(A)** 3D printed PLGA frames are immersed in MSC-suspended PRP solution, and then the gelation of PRP is induced by addition of CaCl_2_ solution with thrombin to complete the CTE construct. Reproduced with permission from Tang Y. et al. (2021). **(B)** Biomaterials, MSCs, and PRP are all mixed together before fabrication process to construct the synergistic scaffold. Reproduced with permission from Wu et al. (2020a).

The clotting ability of activated platelet derivatives expands their application so that they can be applied to the surface of CTE constructures as a coating (Luo et al., 2020), or gel with a 3D printed frame emerging-in as shown in Figure 5A (Tang Y. et al., 2021). In such cases, the platelet derivatives are attached to molded biomaterials, therefore the influences on mechanical and degradation properties are minor. Otherwise, the platelet derivatives are mixed with biomaterials before molding and participate in the structure of CTE as illustrated in Figure 5B. Mild molding conditions are required for activity reservation of platelets or molecules.

Freeze-dried PL is a powdered mixture of GFs and was first incorporated into an electrospun tissue engineering scaffold by (Sell et al., 2011). The addition of the product resulted in uneven fiber diameters and pore areas and decreased mechanical properties. The release of GFs varied from material to material, while all prolonged to more than 35 days. In some strategies with particle materials involved as previously mentioned, platelet derivatives are not only used as bioactive agent sources for biological promotion but also as a material to establish links between the particles and provide at least part of the mechanical support (Santo et al., 2015; Filova et al., 2020; Wu et al., 2021).

When taken into the formula of a hydrogel, the fibrinogen/fibrin could participate in the cross-linked network. Therefore, the physical properties and biological performance of the scaffolds are influenced by the addition of platelet derivatives, roughly according to their form, the blending ratio, characteristic of the biomaterials, and the crosslinking methods. For example, when added into alginate (Alg) hydrogel, with the increase in PRP concentration, the compressive strength and elastic modulus increase, while the degradation is accelerated. In addition, the addition of PRP decreases the gelation rate and forms a more homogeneous structure (Faramarzi et al., 2018; Gao et al., 2019). Similar improvements in mechanical properties and degradation rate are observed in hybrid hydrogels composed of silk fibroin (SF) and PRP, while little variation of gelation time happens, which is mainly correlated with the ratio of poly (ethylene glycol) (PEG) agent and silk concentration (Zheng et al., 2018; Li et al., 2020). 20% (v/v) PRP-mixed 15% (w/v) methacrylated gelatin (Gel MA) hydrogel showed a slight increase in internal pore size and porosity, higher swelling ratio, and lower compressive modulus compared to those without PRP (Jiang et al., 2021). When PRP composed 50% of the hydrogel with a lower Gel MA concentration and was activated before photo-crosslinking, a significant increase in storage modulus, complex modulus, and weight loss were observed (Irmak and Gumusderelioglu, 2021). It is hard to find a pattern as the quality of platelet derivatives, blending ratio, and cross-linking degree vary from formula to formula and factors interact with each other. For example, with some functional groups of polymer chains occupied by electrostatic and hydrogen bonds, the addition of PRP reduces the extent of chemical crosslinking (Khalilijafarabad et al., 2021). In addition, components in platelet derivatives such as catalase could decompose the H_2_O_2,_ thus inhibiting enzymatic crosslinking, and the addition of excess reagent can restore the storage modulus value (Jooybar et al., 2019). In conclusion, the optimum concentration of platelet derivatives is determined not only by their own bioactivity but also by the properties of the whole construct.

#### 3.1.3 Materials of Platelet Derivative Conducting CTE Scaffolds

##### 3.1.3.1 ECM-Based Materials

**FIGURE 6:**
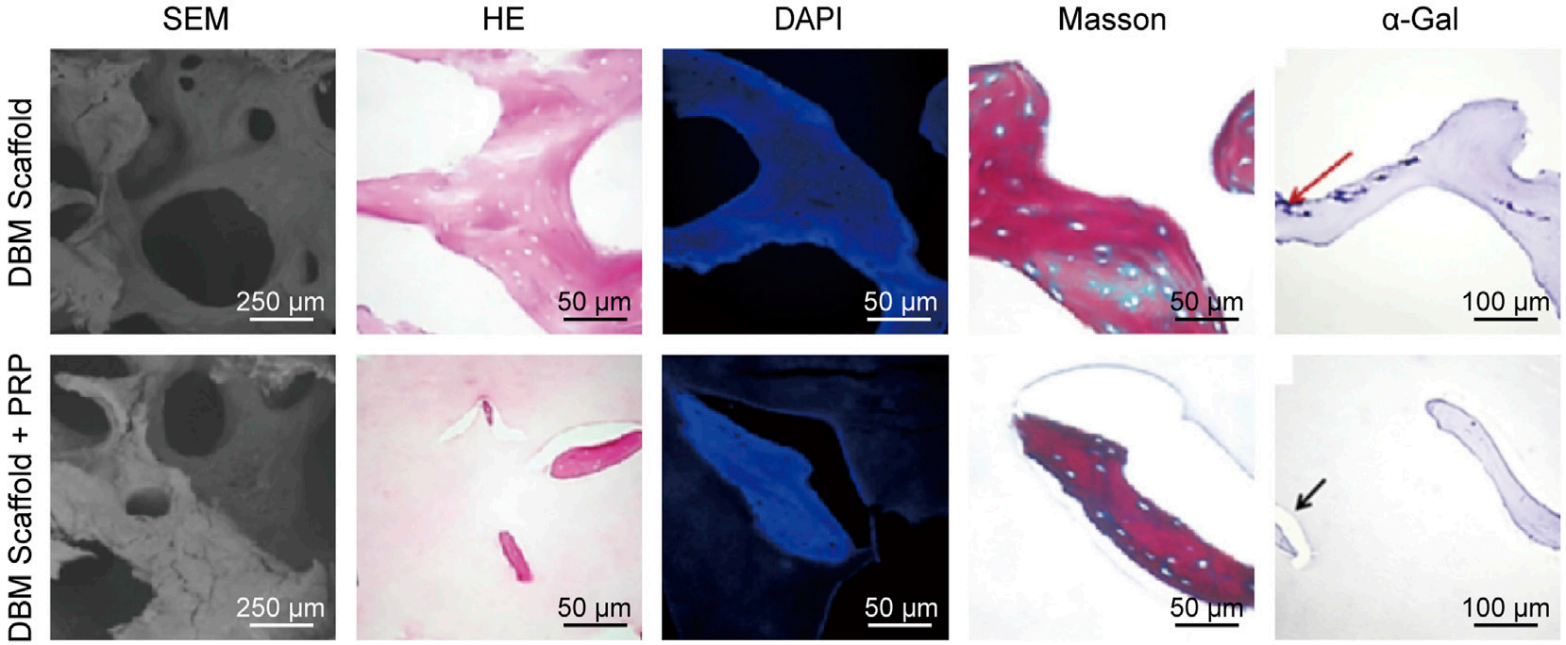
Characterization of decellularized bone matrix scaffolds with PRP. Reproduced with permission from Leng et al. (2020).

ECM is the natural template for synthesized scaffolds, which not only provides structural stability of tissue but also acts as a reservoir of signaling molecules that regulate behaviors and characteristics of the cells within (Figure 6) (Yue, 2014). ECM-based materials are harvested from native cartilage tissue or cultured chondrocytes or MSCs *in vitro*, decellularized with chemicals or devitalized by physical methods to minimize their immunogenicity (Sutherland et al., 2015). With advanced decellularization and devitalization techniques for better mechanical property preservation and recellularization, ECM-based materials appear to be promising for hyaline CTE applications (Huang et al., 2017). In addition, the avascularity and dense nature of articular cartilage may help reduce immune response. So far, xenogeneic decellularized cartilaginous biomaterials have shown good biocompatibility and little excessive rejection (Von Bomhard et al., 2019; Das et al., 2021). Ozdemir et al. loaded allogenic PRP and human BMSCs onto decellularized human placenta scaffolds for treatment of rat osteochondral defects. Though only non-significant improvement by PRP and BMSCs is observed in histological evaluation, evidence of strong chondrocyte proliferation and matrix production and MSCs’ living and differentiation still supports the use of xenogeneic decellularized ECM for cartilage regeneration (Ozdemir et al., 2020).

##### 3.1.3.2 Polymers

Compared to ECM-based materials, polymers are from broader and more abundant sources, with more controllable composition and properties. Natural polymers that exist in the natural ECM of cartilage are popularly used as biomaterials in CTE, including HA, chondroitin sulfate, collagen and its derivative, gelatin (Jiang et al., 2021; Khalilijafarabad et al., 2021). Other natural polymers such as SF, chitosan, agarose, and Alg are also of wide use in tissue engineering. In addition to reliable biocompatibility and biodegradability, biological cues inherent in many natural polymers that promote desirable cell responses also support their use in CTE. Rapid degradation and weak mechanical properties that most natural polymers mutually possess can be remedied with chemical modification or in cooperation with other materials.

Gelatin is a widely used biodegradable and biocompatible protein-based material derived from sources rich in type Ⅰ collagen at a low cost by thermal denaturation and partial hydrolysis (Young et al., 2005). It is suitable for controlled release of bioactive molecules due to polyelectrolyte complexation and degradation itself (Yamamoto et al., 2001). To overcome its instability at body temperature, crosslinking methods are required to form a covalent network. Massive active groups such as –OH, –COOH, –NH_2_, and –SH on the side chains of gelatin are available for functionalization (Sun et al., 2018), and a few are suitable for simultaneous crosslinking and cell encapsulation (Klotz et al., 2016). Of all the methods, photoinitiation provides the best temporal and spatial manipulation over the crosslinking process (Nguyen and West, 2002). Gel MA is the most welcome photo-crosslinking form, with most of the functional amino acid motifs undisturbed and compatible with 3D printing (Yue et al., 2015). Luo et al. 3D printed uniform porous scaffolds with BMSC-laden Gel MA at low concentration of 5% (w/v) for better cell migration and molecule exchange. After 3-week culture, the constructs are evenly applied with freshly activated PRP gel for GFs delivery and implanted intramuscularly in nude mice for another 4 weeks, resulting in hyaline-like tissue with islands of mature chondrocytes dispersed (Luo et al., 2020). As bioprinting is performed in a mild condition where cells can survive, platelet derivatives are able to be components of the bio-ink. Irmak and Gumusderelioglu diluted Gel MA into gradient concentrations with different ratios of PRP, added adipose-derived mesenchymal stem cells (AdMSCs) and gradually 3D printed, photo-activated and cross-linked a tri-layered scaffold to mimic the natural cartilage–interface–bone structure. Constructs are cultured with a mixture of equivalent osteogenic medium and chondrogenic medium in an incubator. AdMSCs were evenly distributed in the osteochondral constructs, differentiated into chondrogenic, hypertrophic, and osteogenic phenotypes based on their location in different phases of the scaffold and formed corresponding tissues *in vitro* (Irmak and Gumusderelioglu, 2021).

HA, chondroitin sulfate (CS) and keratan sulfate are the main GAGs in adult articular cartilage (Kuiper and Sharma, 2015). In particular, various dosage forms of HA and CS are of wide clinical use in OA (Bishnoi et al., 2016; Gupta et al., 2019). Reviews assessing the therapeutic mechanisms of HA indicate that it generally expresses antioxidative, anti-inflammatory, and analgesic effects, and by direct interaction with chondrocytes through particular receptors, it mediates chondroprotective, anti-apoptosis, and cartilage repair effects (Gupta et al., 2019). Similarly, the anti-inflammatory and structure-modifying activities also support the application of CS as a symptomatic slow-acting drug (Bishnoi et al., 2016). As biomaterials, both HA and CS exhibit long-term safety and high biocompatibility for CTE applications. A chemical cross-linked HA/CS/carboxymethyl Chitosan hydrogel was used as a PRP conveyer and induced the process of differentiation of stem cells into cartilage *in vitro*. Through interaction between the aldehyde group of the hydrogel and the amino groups of PRP, it was loaded onto the hydrogel for prolonged release along with the degradation of crosslinking network (Khalilijafarabad et al., 2021). Both HA and CS are negatively charged polymeric molecules available for polyelectrolyte complexation that form particles or aggregates for drug delivery systems (Meka et al., 2017). Santo et al. fabricated CS/Chitosan nanoparticles and PL is loaded through adsorption with an expected burst release of GFs. Through a centrifugation step, the nanoparticles are assembled together, obtaining a hydrogel-like construct that entraps the stem cells in the network. The presence of PL stabilized the construct and held the cells, especially before the deposit of new ECM (Santo et al., 2015).

**FIGURE 7:**
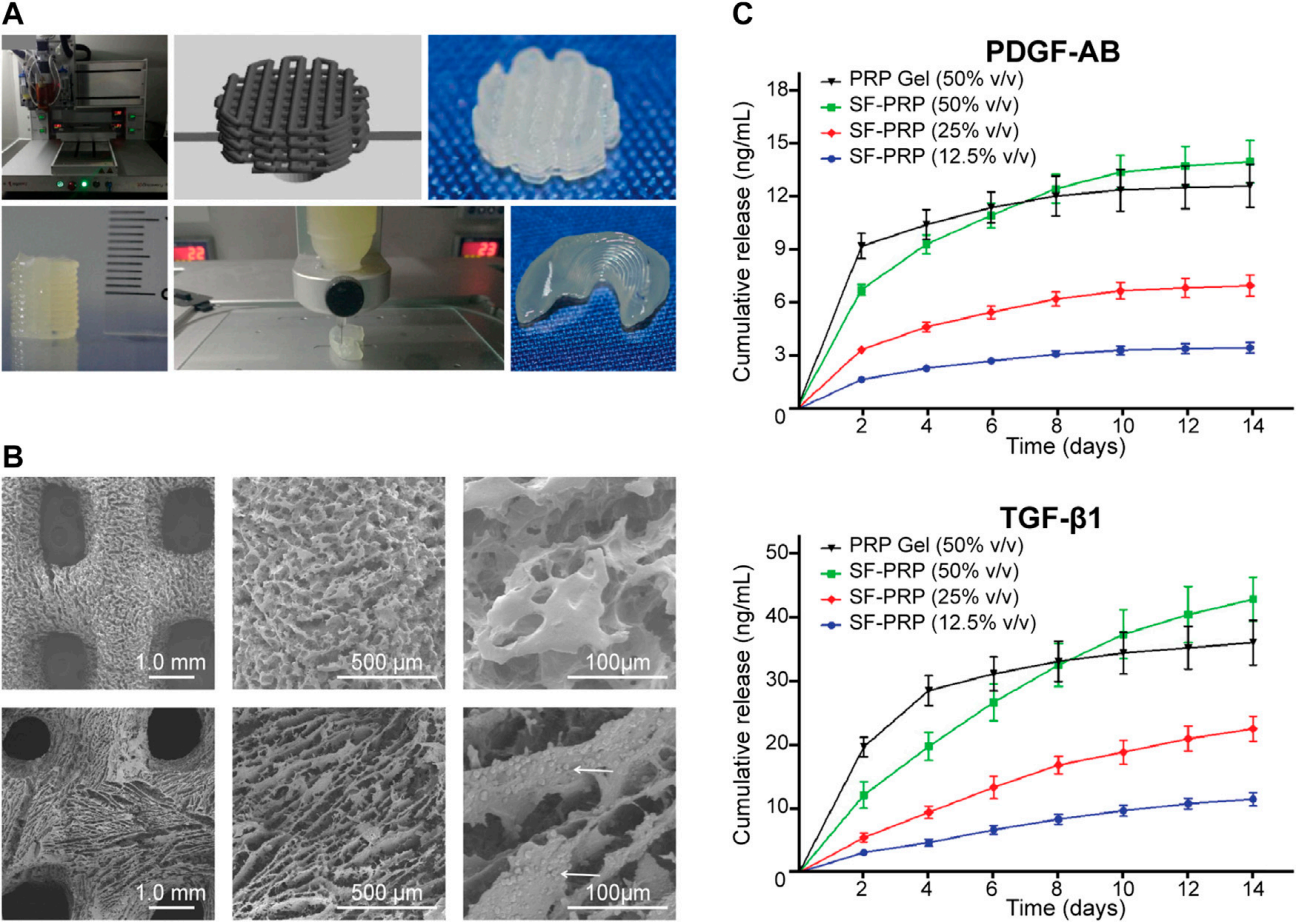
3D bio-printed scaffolds of SF-PRP bio-inks with high resolution, shape fidelity, and controlled release of GFs. **(A)** Three-dimensional printing process of objects with various shapes and dimensions. **(B)** Microstructure of 3D printed scaffolds (white arrows indicate platelets attached in the scaffold). **(C)** Sustained release of GFs from SF-PRP hydrogels. Reproduced with permission from Li et al. (2020).

SF is a protein-based polymer derived from silk produced by insects such as silkworms and spiders. It is immunologically inert and possesses low inflammatory potential, purified by complete removal of potential inflammation-inducing proteins and debris (Kundu et al., 2014). The degradation rate depends on the methods of isolation and reconstruction process of the material, the different formats it’s fabricated into, and the abundance of enzymes in the implantation site (Debari et al., 2021). Bio-ink composed of SF and PRP is 3D-printed to scaffolds and cross linked with PEG. With a cumulative GF release increase compared to thrombin-activated PRP gel, the GFs were almost linearly released for 14 days (Figure 7) (Li et al., 2020).

Compared to some proteins, polysaccharides do not suffer from some of the disadvantages, such as immunogenicity and potential risk of animal-originated pathogen transmission. Chitosan is a natural polysaccharide derived from chitin, a structural element in the exoskeleton of crustacean, which is easily extracted from the protective shells of crabs and shrimp (Hamed et al., 2016). It is distinctive due to the cationic nature capable of polyelectrolyte complexes forming and its broad use in drug delivery and ECM mim icking (Bhattarai et al., 2010; Zhang Z. et al., 2021).

Agarose is the unmodified neutral backbone of agar, extracted from cell walls of algae, that can self-assemble into a thermal-reversible gel (Michel et al., 2006). Its mechanical behaviors are easily modified by concentration to mimic articular cartilage upon static or dynamic loading (Salati et al., 2020). Lacking integrin binding sites, agarose shows less promotion of cell adhesion compared to collagen, but exceeds in cellular metabolism and biosynthesis of chondrocytes (Guaccio et al., 2008). Yin et al. fabricated PRP–agarose gels encapsulated with chondrocytes via physical blend and accelerated cell proliferation in the early stage of *in vitro* cultivation (Yin et al., 2014). Agarose exhibits neutral surface charge at different pH that results in weak electrostatic forces for GFs adsorption and reservation, thus chemical modification and copolymerization with other polymers are recommended for sustained release (Yazdi et al., 2020).

Alginic acid is another abundant marine bio-polymer, extracted from brown seaweed. Its linear structure is composed of alternating blocks of 1–4 linked α-L-guluronic and β-D-mannuronic acid residues, and the stacking of guluronic-blocks with divalent cations such as Ca^2+^ presence causes gelation and cross-linking under mild conditions (George and Abraham, 2006). As previously mentioned, calcium ion also triggers platelet degranulation and coagulation cascades, and therefore the gelation agent matches and forms a more homogeneous structure (Gao et al., 2019). Similar to agarose, pure Alg is short of cell adhesion signaling moieties and the blending of adhesive proteins, such as fibrinogen in platelet derivatives, mends the drawback and provides extra promotion for rich in GFs.

Synthetic polymers are attractive candidates for CTE for typically more flexible and controllable mechanical and chemical properties than those of natural materials, readily available, and relatively low cost of industrial scale production. Poly (α-hydroxy) acids are the most widely used synthetic degradable polymers such as polycaprolactone, poly (glycolic acid), poly (lactic acid), and their copolymers (Place et al., 2009). Bioresorbable devices made from aliphatic polyesters are now used worldwide in surgery and controlled drug delivery (Albertsson and Varma, 2003). Platelet derivatives can be loaded onto such commercialized scaffolds by methods as simple as immersing (Kreuz et al., 2013). Their simplicity compared to protein-based polymers leaves them with low immunogenicity but also limited bioactivity. Chemical modification is an important method of functionalization, but the addition of platelet derivatives is an easier way. Tang et al. 3D printed the frame with poly (lactide-co-glycolide) (PLGA) and laden BMSCs and PRP onto the scaffold in one step though induced gelation. The scaffold had been completely absorbed 8 weeks after the implantation (Tang Y. et al., 2021). Zhang et al. manufactured bilayer PLGA scaffolds with different pore sizes by compression molding and particulate leaching. BMSCs are seeded onto the scaffold and incubated for attachment, and PRP is loaded prior to implantation surgery. The addition of platelet derivatives resulted in better cartilage repair, and BMSCs enhanced osteogenesis of subchondral bone, while the PLGA scaffolds have not been completely degraded 6 months after the implantation (Zhang et al., 2015). Elasticity shock absorbent material is a direction for mechanical modification of synthetic polymers that physically mimics the natural ECM of chondrocytes, and addition of platelet derivatives serves as a biological improvement. Rothan et al. structured polycaprolactone triol–citrate with the solvent-casting/particulate leaching method, soaked in soluble platelet releasates and freeze-dried to produce an elastomeric porous scaffold (Rothan et al., 2017).

##### 3.1.3.3 Inorganic Materials

Inorganic materials, including metallic materials, bioactive glasses, and bio-ceramics, are widely used in bone tissue engineering, for their load-bearing ability (Collins et al., 2021). As one of two nourishing pathways, subchondral bone plays an important role in cartilage regeneration and influences the quality of regenerated tissue (Nishitani et al., 2009; Zhang S.-Y. et al., 2021). In addition, a stronger structure for the subchondral bone layer could reduce the shear forces and benefit the integration of the cartilage layer under dynamic conditions. Thus, the introduction of bone-mimicking materials for subchondral bone reconstruction in a biphasic or multiphasic scaffold has drawn a lot of attention.

Of all the inorganic materials for bone reconstruction, calcium phosphate ceramics hold the most similarity to the mineral components of bone. By chemical compositions and crystal phases, they are classified into different types, among which hydroxyapatite (HAP), beta-tricalcium phosphate (β-TCP), and their mixture known as biphasic calcium phosphate. In addition structure-based osteoconductivity, they also serve as a source of calcium ions for ossification through surface dissolution (Tang et al., 2018). They have been studied extensively and put into clinical use in bone substitution, reconstruction, and drug delivery (Ragaey and Van Sickels, 2017; Maenhoudt et al., 2018; Zhao et al., 2020). The porous structure of the materials also provides good absorption of liquid state platelet derivatives. Calcium polyphosphate substrates seeded with chondrocytes were used as bio-engineered cartilage in an *in vitro* model for study of integration (Theodoropoulos et al., 2011). Soaking in PRP before press-fit implantation increased expression of matrix degrading/remodeling genes at the interface and strengthened the integration to the host (Sermer et al., 2018). Jang et al. immersed cylinders of HAP into platelet-rich fibrin glue with MSCs and left a 2 mm depth of hydrogel as the cartilage layer. The platelet derivatives serve as cell-seeding media, the scaffold of the cartilage layer, and bioactive-molecule resources for both layers (Jang et al., 2014).

Combination of inorganic materials and polymers unites their advantages in osteochondral regeneration. With the addition of nano-HAP, the compressive strength was increased by more than two-fold compared to the CS/SF scaffold, with minor influence on degradation rate and facilitated cell adhesion and proliferation (Qi et al., 2014). PRP and lentivirus-transduced BMSCs with stable expression of BMP-2 was further loaded on the CS/SF/nano-HAP scaffold and provided promising results *in vivo*. In particular, the BMP-2 expression is significantly increased with the treatment of PRP, which indicates platelet derivatives as stimulators of transferred genes (Ruan et al., 2018). As most of the GFs in platelet derivatives are basic, acidic gelatin showed increased sorption through the formation of poly-ion complexes (Yamamoto et al., 2001). Seo et al. used an acidic gelatin β-TCP sponge as a carrier of PRP for cartilage and a basic sponge with BMP-2 for bone regeneration, and implanted MSC-loaded constructs sequentially into the osteochondral defect (Seo et al., 2013).

### 3.2 Other Usage of Platelet Derivatives in CTE

There are approaches to extended use of platelet derivatives other than scaffold ingredients for bioactive upgrade and structural unity. For example, Brehm et al. use autologous PRP gel as an adhesive between the graft and the subchondral bone yet get little improvement in retention of the implant because of its poor mechanical strength and adhesion force (Brehm et al., 2006).

Successful attempts generally take advantage of bioactive molecules they content. As previously mentioned, platelet derivatives can be used as a culture medium supplement for CTE constructs before implantation for better cell proliferation, differentiation, and ECM accumulation (Petrera et al., 2013). As the metabolic activation of cells will not fade away so quickly and the reservation of active components by the scaffold, the improvement of cartilage formation could last after the implantation and improve the integrity of regenerated tissue to the surrounding tissue (Van Pham et al., 2013; Beigi et al., 2018).

Derived from PRP treatment of knee OA, intra-articular platelet derivatives can be used as treatment adjuvants for improvement after transplantation. Once per 2 weeks intra-articular injection of PRP enhanced BMSC-laden collagen scaffold implantation, resulted in a higher level of chondrogenic differentiation markers and historical score (Fang et al., 2019). Dominguez Perez et al. treated full-thickness articular cartilage defects of sheep with implantation of an autologous-based matrix of hyaline cartilage chips combined with plasma clot and intraarticular injection of PRP, resulting in hyaline cartilage regeneration and integration with surrounding margin at 6 months, with a similar structure and collagen expression pattern compared to the healthy adjacent cartilage (Dominguez Perez et al., 2019).

## 4 Conclusion and Perspectives

Biological is a complex mixture of numerous bioactive molecules ideally functional in tissue regeneration. Although most of the main components of platelet derivatives have been independently studied in cartilage repair, the content of individual bioactive molecule varies depending on the protocol of extraction, format of the product, process of activation, and even health condition of the donor. Elucidation of the molecular complexity of these products and determining the dosing for specific tissue regeneration remain great challenges. The combination with biomaterials sustains the release of bioactive molecules and has made it even more complicated that both temporal and spatial distribution should be considered to meet the needs of developing CTE tissue and cells. Removal of defect components in platelet derivatives such as VEGF in cartilage diseases might be realized by “filter-like” advanced biomaterials or through an optimized extraction process (Zhu et al., 2013; Hamilton et al., 2016). Recent studies have emphasized mechanical stimulation and mechanical properties of the scaffold (Zhang and Yao, 2021), because the stem cell lineages are largely specified by matrix elasticity, in spite of the addition of soluble induction factors (Engler et al., 2006). According to these theories, the addition of platelet derivatives initiates the proliferation and differentiation of the stem cells, while the proper scaffold manufactured and the microenvironment the cell makes for itself will ultimately determine its destiny. Unlike most of the soluble factors, the addition of platelet derivatives sometimes influences the mechanical properties and degradation of the CTE construct. In those situations, they should be considered as a hybrid material rather than an additive AND a material. In addition, most of the studies evaluate the osteochondral regeneration by histological, histochemical, and biomechanical analysis. Although articular injection of PRP may not be as effective as previously thought in knee OA (Bennell et al., 2021), the improvement of motor function and alleviation of pain is still the ultimate purpose of the preclinical treatment of CTE and needs to be evaluated in follow-up studies.

In conclusion, the combination of platelet derivatives and biomaterials takes full advantage of the safe and low-cost resource of bioactive molecules that have been proved to accelerate cartilage regeneration and integration of neo-tissue and the origin parts. As variability is a common defect in biologicals, detailed information about platelet derivatives is required to maximize reproducibility of results. Further research of platelet derivatives in combination with biomaterials should aim to realize spatiotemporal delivery to meet the regeneration of articular cartilage, restore the function of the joint, and ultimately improve patients’ quality of life.

## Abbreviations

ACI, autologous chondrocytes implantation; AdMSC, adipose-derived mesenchymal stem cell; Alg, alginate; BMP, bone morphogenetic protein; BMSC, bone marrow mesenchymal stem cell; β-TCP, beta-tricalcium phosphate; CPC, chondrogenic progenitor cell; CS, chondroitin sulfate; CTE, cartilage tissue engineering; ECM, extracellular matrix; GAG, glycosaminoglycan; Gel MA, methacrylated gelatin; GF, growth factor; HA, hyaluronic acid; HAP, hydroxyapatite; MSC, mesenchymal stem cell; OA, osteoarthritis; OAT, osteochondral autograft transplantation; PEG, poly(ethylene glycol); PL, platelet lysate; PLGA, poly(lactide-co-glycolide); PRF, platelet-rich fibrin; PRP, platelet-rich plasma; SF, silk fibroin; TGF-β, transforming growth factor beta; VEGF, vascular endothelial growth factor; 3D, three-dimensional.
